# Expediting knowledge acquisition by a web framework for Knowledge Graph Exploration and Visualization (KGEV): case studies on COVID-19 and Human Phenotype Ontology

**DOI:** 10.1186/s12911-022-01848-z

**Published:** 2022-06-02

**Authors:** Jacqueline Peng, David Xu, Ryan Lee, Siwei Xu, Yunyun Zhou, Kai Wang

**Affiliations:** 1 Raymond G. Perelman Center for Cellular and Molecular Therapeutics, Children’s Hospital of Philadelphia, Philadelphia, PA 19104 USA; 2 School of Arts and Sciences, University of Pennsylvania, Philadelphia, PA 19104 USA; 3 College of Arts and Sciences, Emory University, Atlanta, GA 30322 USA; 4 Department of Pathology and Laboratory Medicine, University of Pennsylvania Perelman School of Medicine, Philadelphia, PA 19104 USA

**Keywords:** Knowledge graph, COVID-19, Information extraction, Data visualization, Knowledge discovery

## Abstract

**Background:**

Knowledges graphs (KGs) serve as a convenient framework for structuring knowledge. A number of computational methods have been developed to generate KGs from biomedical literature and use them for downstream tasks such as link prediction and question answering. However, there is a lack of computational tools or web frameworks to support the exploration and visualization of the KG themselves, which would facilitate interactive knowledge discovery and formulation of novel biological hypotheses.

**Method:**

We developed a web framework for Knowledge Graph Exploration and Visualization (KGEV), to construct and visualize KGs in five stages: triple extraction, triple filtration, metadata preparation, knowledge integration, and graph database preparation. The application has convenient user interface tools, such as node and edge search and filtering, data source filtering, neighborhood retrieval, and shortest path calculation, that work by querying a backend graph database. Unlike other KGs, our framework allows fast retrieval of relevant texts supporting the relationships in the KG, thus allowing human reviewers to judge the reliability of the knowledge extracted.

**Results:**

We demonstrated a case study of using the KGEV framework to perform research on COVID-19. The COVID-19 pandemic resulted in an explosion of relevant literature, making it challenging to make full use of the vast and heterogenous sources of information. We generated a COVID-19 KG with heterogenous information, including literature information from the CORD-19 dataset, as well as other existing knowledge from eight data sources. We showed the utility of KGEV in three intuitive case studies to explore and query knowledge on COVID-19. A demo of this web application can be accessed at http://covid19nlp.wglab.org. Finally, we also demonstrated a turn-key adaption of the KGEV framework to study clinical phenotypic presentation of human diseases by Human Phenotype Ontology (HPO), illustrating the versatility of the framework.

**Conclusion:**

In an era of literature explosion, the KGEV framework can be applied to many emerging diseases to support structured navigation of the vast amount of newly published biomedical literature and other existing biological knowledge in various databases. It can be also used as a general-purpose tool to explore and query gene-phenotype-disease-drug relationships interactively.

**Supplementary Information:**

The online version contains supplementary material available at 10.1186/s12911-022-01848-z.

## Background

The impact of emerging diseases on public health has mandated an “all hands-on deck” scientific response [1–4]. Machine learning and artificial Intelligence (AI) offer a means to rapidly generate and update knowledge from the exploding amounts of data from various scientific domains, including scientific literature, social networks, and high-throughput screening experiments. AI technologies, such as natural language processing (NLP), can help detect, predict, and facilitate a better understanding on human diseases [5–7]. However, one major limitation of existing approaches is the lack of open-source and freely accessible tools for use by the broader biomedical research community. They may also suffer from the inclusion of non-informative generic information, due to their accumulation of noisy terms in the absence of rigorous filtering, if focusing on specific scientific domains. In short, current literature mining tools for biomedical text curation are far from optimal; improved and open-source tools to exploit the rapidly growing, unstructured scientific literature to study complex clinical characteristics of emerging diseases are urgently needed [8–10].

To improve the knowledge discovery on emerging diseases, various research groups have started to leverage knowledge graphs (KGs), which help users better understand the relationships among biomedical concept entities (e.g. molecules, organs, genes, drugs, and diseases) from scientific literature and available databases. To begin with, a KG consists of various entities joined by relationships. Entities, regarded as nodes, can represent objects, individuals, or abstract ideas, whereas relations, regarded as edges, represent the nature of connection between nodes. Beyond a KG’s utility in simplifying information into entities and relations, KG embedding, visualization, and reasoning can be used for further knowledge acquisition [11–14]. When applied to biomedical domains, a KG can contain deep-curated connections—from molecular mechanisms to phenotypic manifestations of human diseases—to support hypothesis generation, hypothesis validation, and knowledge discovery in research studies. For example, curated relationships may include simple co-occurrence relationships such as known drug-disease associations, disease-disease co-morbidity, and protein–protein interactions; they may also include more complex and descriptive relationships, such as ‘TREATS’, ‘INHIBITS’, and ‘CAUSES’, using semantic networks.

Beyond literature, biomedical researchers also have access to annotated relationships from various biomedical knowledgebases and ontologies, such as protein–protein interactions from STRING [15] and drug-target relationships from DrugCentral [16], to create more comprehensive KGs [17]. Downstream of their creation, KGs have been used to predict re-purposable drugs for a disease based on KG completion [18], generate drug repurposing reports [19], as well as facilitate the retrieval of scientific articles to answer questions based on the KG [20]. With relevant research continuously evolving, the KG framework will serve an effective tool to organize overwhelming scientific findings in a structured format that can advance scientific research based on currently available knowledge.

Taking the global pandemic of coronavirus disease COVID-19 as an example, the academic community has raced to publish scientific findings about the disease [21]. Because of the literature explosion, researchers are eager to be informed with the most relevant, focused, and conclusive findings from the massive amounts of COVID-19 literature generated within a short period of time [22–24]. While many research efforts have been focused on improving the construction of KGs about COVID-19 [17, 25, 26], there is less emphasis on the exploration and visualization of KGs for knowledge discovery, which is the case for COVID-19 as well [27]. For example, few COVID-19 KG papers have developed publicly accessible tools that allow direct and interactive exploration over the KG, as opposed to running queries over it and returning results. Furthermore, few open-source tools have been developed specifically for the exploration and visualization of continuously updated KGs, despite the presence of many web services that serve static KGs generated by various means.

In this paper, we outline an open-source framework called Knowledge Graph Exploration and Visualization (KGEV), for interacting with KGs as a web application using React as the frontend, Flask as the backend, and Neo4j as the graph database. The framework facilitates the composition of KG data through the integration of relationships from literature with relationships from the various ontologies and databases, the formatting of the KG data for Neo4j import, and the deployment of the web application on a user’s own web server (or even a user’s own laptop). The web application itself allows direct exploration of the COVID-19 KG through an intuitive user search interface, as well as tools such as shortest path detection, fuzzy search, and various node and edge filters. Moreover, in addition to visualizing the COVID-19 KG, literature evidence supporting relationships in the KG can be viewed and searched as well, making links in the KG more interpretable. Overall, our proposed framework supports KG visualization, data exploration, and hypothesis generation in a user-friendly manner. Although we demonstrate the powerful usage of our tool using a COVID-19 KG as a case study, the framework can be, and was intentionally designed to be, easily extensible to KGs for other emerging diseases or even general-purpose KGs. To support this, we demonstrate a turn-key adaption of the KGEV framework to study clinical phenotypic presentations of human diseases, illustrating the versatility of the framework.

## Methods

### Literature data sources and processing procedure

The COVID-19 Open Research Dataset (CORD-19) [28] version 82 (last updated 2021–03-08), which is available publicly at https://www.kaggle.com/allen-institute-for-ai/CORD-19-research-challenge, was used as the source from which information was extracted for the first stage of KGEV. Only text from abstracts were used. SemRep [29], a natural language processing tool used to mine triples (head, relationship, tail) from biomedical texts, was applied on the abstracts from CORD-19. SemRep version 1.8 was used with default settings. SemRep was also used to normalize entities to concepts from the UMLS Metathesaurus using MetaMap 2018AB [30]. Only triples where both the head and tail entity had a confidence score of at least 800, as determined by SemRep, and appeared more than once were used. All SemRep relationship types were used with “INFER” and “SPEC” specifications removed as these relationship types are inferred relationships based on the other generated SemRep predictions. All SemRep semantic types were used and they were mapped to their respective semantic groups.

Since SemRep was not trained to recognize COVID-19-/SARS-CoV-2-specific terminology, previously curated dictionaries [31] representing the concepts of COVID-19 and SARS-CoV-2 were used to standardize these terminologies. Specifically, these dictionaries were used to extract COVID-19-/SARS-CoV-2-related terminology using a string-matching approach on original texts detected by SemRep. Then, these terms were manually reviewed and mapped onto a small number of normalized COVID-19-/SARS-CoV-2-related subjects, which are specified in Additional file 1: Table S1.

### Additional data sources from public databases

In addition to CORD-19, several other data sources were integrated into the KG (Additional file 2: Fig. S1). Disease-gene relationships were obtained from DisGeNET [32], drug-gene relationships were obtained from DGIdb [33], gene–gene/protein–protein relationships were obtained from STRING [15], gene/protein-gene ontology [34] (GO) relationships were obtained from Uniprot [35], and disease-phenotype and disease-gene relationships were obtained from the Human Phenotype Ontology [36] (HPO).

To integrate the relationships from these public data sources into the KG generated from CORD-19 triples, only relationships where both entities already exist in the KG were considered. Therefore, incorporating additional data sources is essentially done by adding additional edges between nodes in the KG. In order to perform this integration, entities in a data source need to be mapped to nodes in the KG. This node mapping is done by first mapping data source entities to their UMLS Concept Unique Identifier (CUI), using files from HGNC [37], Disease Ontology [38], and UMLS [39], and then mapping this CUI to the CUI of the KG nodes, which were outputted by SemRep in the CORD-19 relationship extraction step. The KGEV pipeline provides the flexibility to integrate on other node/entity attributes besides CUI.

### KGEV pipeline

**Fig. 1:**
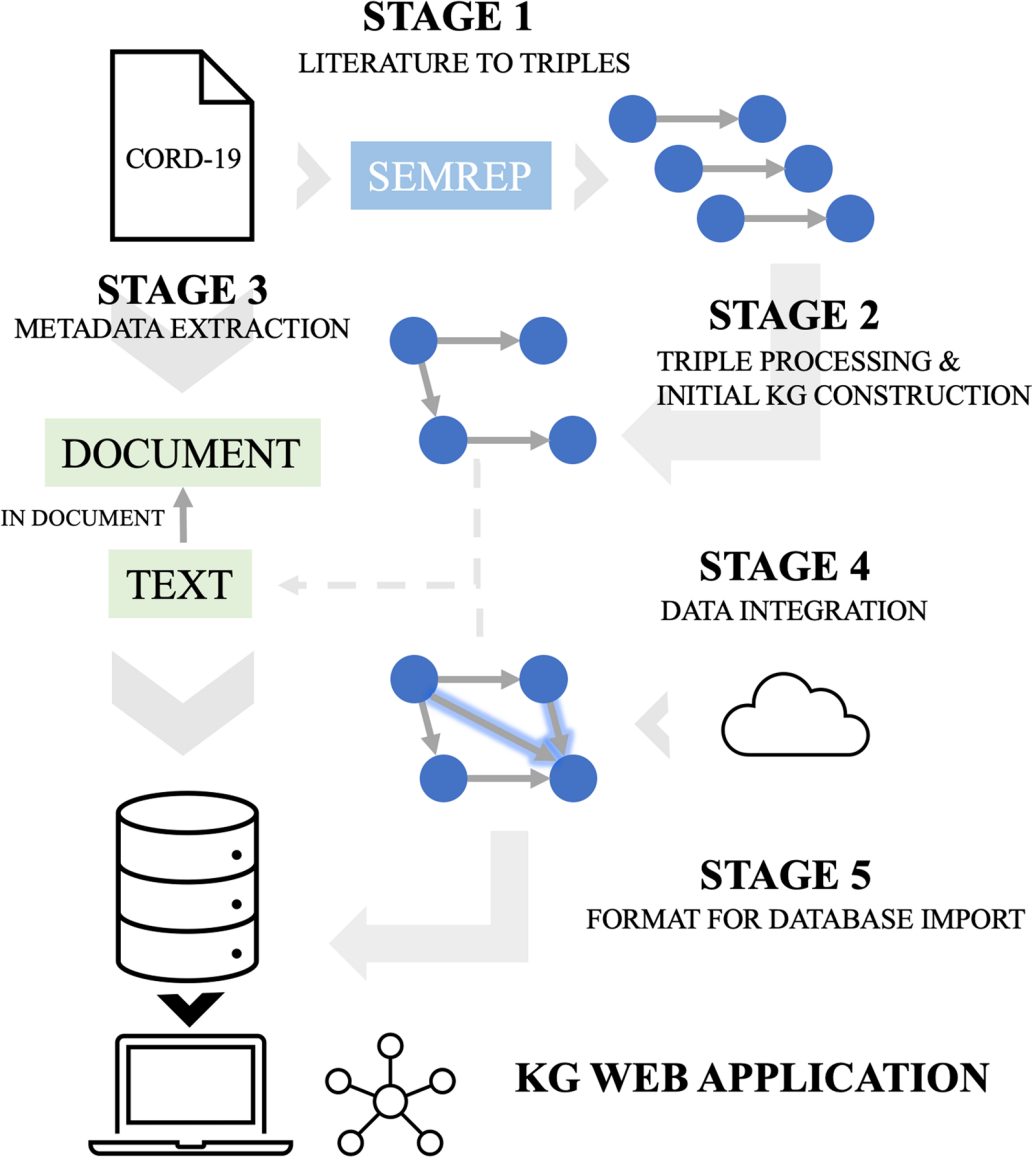
KGEV framework stages. Stage 1: A literature mining tool is used to extract relationships from texts such as biomedical literature or clinical notes to produce triples (a head and tail entity linked by a relationship). In our COVID-19 case study, we used SemRep to extract triples from CORD-19 abstracts, which contains literature related to COVID-19. Stage 2: The extracted triples are filtered to keep only high-quality triples. In our case, we filtered by the SemRep confidence score and the number of times a triple appears in the data. We also normalized COVID-19-/SARS-CoV-2-related entities to a small number of topics. The triples from this step are used to construct the initial knowledge graph. Stage 3: Metadata is extracted from the texts that the triples originated from. This metadata, which includes information on the immediate text that supports the extracted triple and document-level information, is stored in a database. Stage 4: Data from other sources are integrated into the KG to fill in the gaps of the triples mined from the texts. In our COVID-19 case study we used eight additional data sources (see Methods for details). Stage 5: The integrated triples are then formatted and stored in the database along with the metadata information**.** In our case, we used Neo4j as the database because its graphical representation of the data naturally fits the KG data structure

The KGEV pipeline involves five stages: triple extraction, triple filtration, metadata preparation, knowledge integration, and graph database preparation (Fig. 1). Briefly, the first stage involves extracting triples from biomedical literature, and the second stage involves filtering these triples and constructing the initial knowledge graph. The third stage involves extracting metadata for the triples. The fourth stage involves integrating triples from other data sources. The fifth and final stage is formatting the knowledge graph for import into the web server’s database. These stages are described in more detail, and how they work for the COVID-19 use case, in the Results section, in the “COVID-19 as a use case for knowledge integration and KG development” subsection.

### Development of web application

Our web application was built using three modern frameworks, which are React, Flask, and Neo4j. The frontend of our web application uses the React framework, an open-source frontend JavaScript library for building user interfaces that is maintained by Facebook. In order to display the graph, we used cytoscape-js along with its React component, react-cytoscapejs. cytoscape-js is an open-source graph/network JavaScript library for network visualization and analysis. The backend of our web application was developed using Flask, a web framework written in Python. It connects to the Neo4j graph database, which stores the KG data, using the py2neo Python package, a client library for working with Neo4j through a Python interface. We use Neo4j’s Cypher query language to run queries over the Neo4j data. Full-text indexing of nodes in the Neo4j database was used for efficient searching. Neo4j-admin was used to import the triples data, text data, and article metadata into the Neo4j database. The web application is deployed as two Docker containers, one for the backend application and one for the frontend application.

## Results

### Overview of the Knowledge Graph Exploration and Visualization (KGEV) framework

In our framework for knowledge graph exploration and visualization (KGEV), the knowledge graph (KG) can be developed in five stages: triple extraction, triple filtration, metadata preparation, knowledge integration, and graph database preparation (Fig. 1). The first stage involves triple extraction from biomedical texts (such as published literature on a specific disease, or all clinical notes on patients with a specific disease) using a natural language processing tool. The second stage involves filtering the triples to obtain high-quality triples. The third stage involves extracting the high-quality triples’ metadata and formatting the data for import into the web application database. Depending on the number of texts used in the first stage, the resulting KG may be sparse (i.e. some entities may be mentioned only once, and some true edges between KG nodes may be missing). Therefore, the fourth stage involves integrations of relationships from other data sources, such as public databases, that have relationships between different entities in the KG. The fifth and final stage involves formatting the KG nodes and edges for import into the graph database to be used in the web application. We selected Neo4j as the graph database engine in our framework, because it is a transactional database with native graph storage and processing, and it is available in an open-source “community edition” in addition to the commercial edition.

**Fig. 2:**
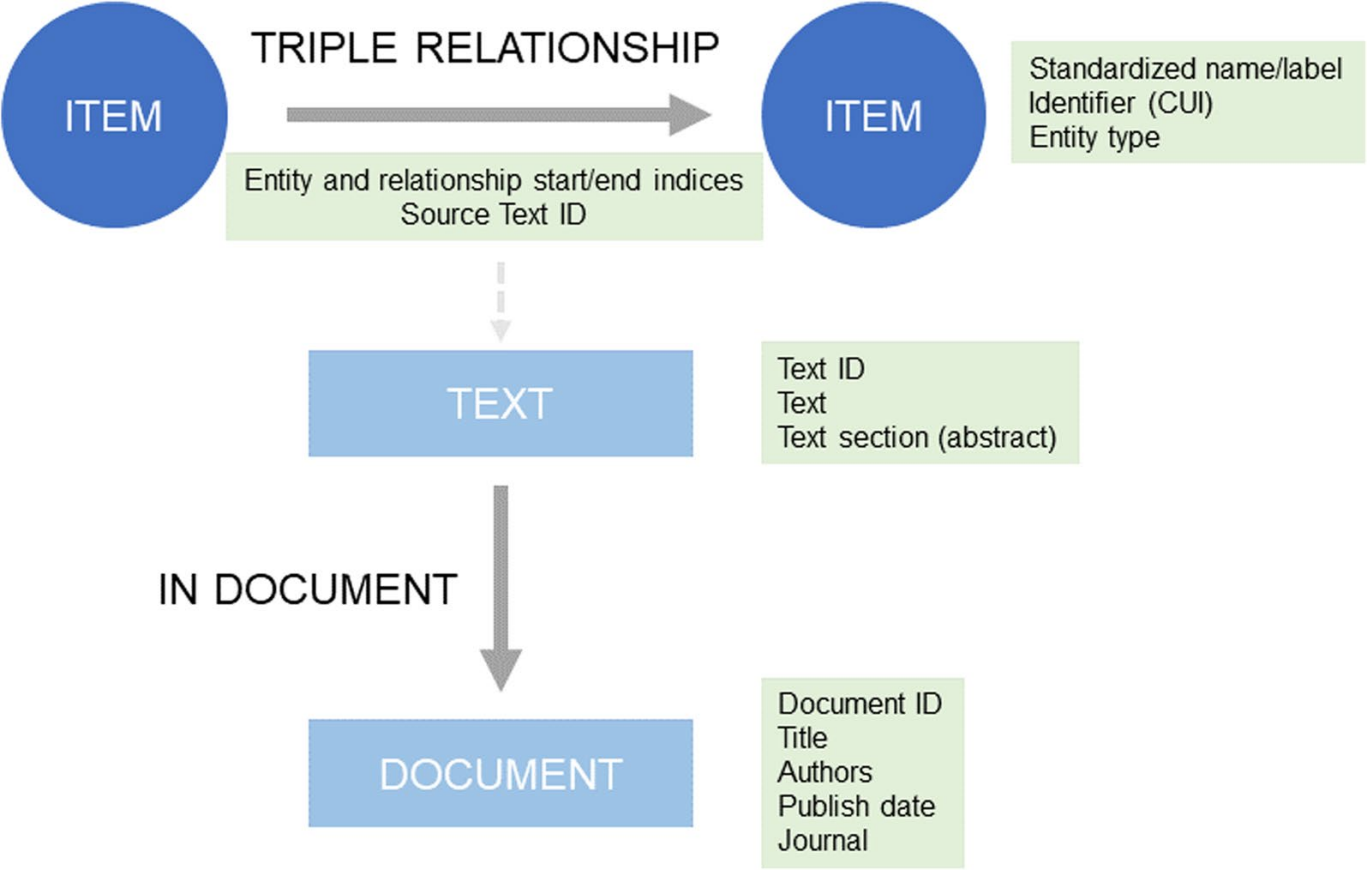
Database schema for KGEV. The database has three types of entities: ITEMs, TEXTs, and DOCUMENTs. The properties stored for each item type are shown in the green panels. The “IN DOCUMENT” relationship connects a TEXT item (e.g. article sentence) to its corresponding DOCUMENT (e.g. article). ITEMs are connected through SemRep relationships (TREATS, CAUSES, INTERACTS_WITH, etc.) in our COVID-19 example. Given a SemRep triple (ITEM—> SEMREP RELATIONSHIP—> ITEM), corresponding/supporting texts can be retrieved by joining the Source Text ID property of the triple’s edge to the TEXT entity with that ID

Importantly, the pipeline and source code were designed for adaptive use. For example, for the first step, any NLP software tool that extracts relationships from text could be used, and the rest of the KG development pipeline would still function. Additionally, the data sources for the fourth stage of the pipeline could be expanded as new data or knowledgebases becomes available or re-integrated as the data quality increases. Since the web application was designed so the KG content only depends on the graph database, as long as the data in the database are in the correct schema (Fig. 2), different KGs can be swapped in and out, with a simple change of the configuration to use a different Neo4j graph database. The adaptivity and scalability of the pipeline means that it can be applied to many different biomedical domains. In the sections below, we used COVID-19 as a case study to illustrate how the framework can help explore existing knowledge from literature and public databases, because COVID-19 is an emerging disease with a strong practical need for users to explore the vast amount of knowledge published within a short period of time. Furthermore, we also demonstrated a turn-key adaption of the KGEV framework to study clinical phenotypic presentation of human diseases, illustrating the versatility of the framework.

### COVID-19 as a use case for knowledge integration and KG development

As a case study, here we describe how we used the five-stage procedure to study COVID-19 using the KGEV framework (Fig. 1). Rather than searching for papers relevant to COVID-19 ourselves from PubMed or bioRxiv/medRxiv preprint servers, we decided to use the COVID-19 Open Research Dataset (CORD-19) instead [28]. As described in its website (https://www.kaggle.com/allen-institute-for-ai/CORD-19-research-challenge), CORD-19 is a continuously updated resource of scholarly articles about COVID-19, SARS-CoV-2, and related coronaviruses. This freely available dataset is provided to the global research community to apply recent advances in NLP and other AI techniques to generate new insights in support of the ongoing fight against this infectious disease. In the current study, we used SemRep [29], a natural language processing tool used to mine triples, to process the CORD-19 data and generate triples in the first stage. We ran SemRep on all abstracts from CORD-19 version 82 (last updated 2021–03-08), and in total the dataset contained 309,175 unique abstracts, including 170,722 abstracts with PMC IDs, and 233,312 abstracts with PubMed IDs. SemRep initially discovered a total of 1,123,654 relationships, which consist of 50,185 unique entities normalized to Unified Medical Language System (UMLS), 31 unique relationship types, and 373,327 unique triples.

In the second stage, we performed filtration of the triples to reduce the number of triples in subsequent steps. After keeping only high-confidence relationships and entities, the number of triples was reduced to 590,923, including 96,108 unique triples, 21,694 unique entities, and 31 unique relationship types. The triples were used to construct a KG, where the nodes are the head and tail entities from the triples and the edges are the relationships between them. A total of 273 unique COVID-19-/SARS-CoV-2-related entities were identified among the filtered SemRep triples, including 51 entities that were normalized to “COVID-19” and 21 entities that were normalized to “SARS-CoV-2” (Additional file 1: Table S1).

In the third stage, metadata information was collected from the CORD-19 dataset. For each triple extracted from CORD-19, the following related metadata fields were collected: the supporting text/sentence from which the triple was directly extracted from, the CORD-19 ID of the article the triple came from, and the authors, title, journal, and publish date of the article. Additionally, we also downloaded the latest information on journal impact factors from SCI Journal Citation Reports, and integrated them as part of the metadata, because such information may sometimes be helpful to judge the reliability of knowledge presented in biomedical texts. These data were imported into the web application database such that unique supporting texts were stored separately from triples, so they would not need to be loaded into memory unless a specific triple was queried. Moreover, articles were also stored separately with links from supporting texts to articles, since multiple texts can come from the same article (Fig. 2). We emphasize that this is a major difference between our KGEV framework and other KG on COVID-19: for each triple relationship, we have the supporting text/sentence from which the triple was directly extracted from. Therefore, a human reviewer can read the supporting texts and decide whether the relationships are trustworthy based on their contexts in the biomedical literature. In other KGs on COVID-19, although a confidence score can be assigned to a specific relationship, users typically do not have the opportunity to review a paragraph of scientific text to judge whether the relationship is reliable.

In the fourth stage, integration of other data sources was performed. Although the CORD-19 dataset contains a very large number of abstracts that summarize recent findings, the KG created from solely CORD-19 abstract triples can be relatively sparse. While COVID-19 related entities are likely to be mentioned at least once in CORD-19, and therefore appear as nodes in the KG, some true edges between KG nodes may be missing because of the limited size of the dataset. Therefore, we integrated knowledge of entity relationships from five other data sources: the Drug Gene Interaction Database [33], DisGeNET [32], Human Phenotype Ontology (HPO) [36], STRING protein–protein interaction database [15], and Uniprot [35]/Gene Ontology (GO) [34], in addition to using HGNC [37], Disease Ontology [38], and UMLS [39] for entity standardization. These relationships were integrated by matching on the nodes obtained from CORD-19 triples and adding additional triples/relationships to the existing nodes, where the relationship type is disease-gene, drug-gene, etc. In total we integrated 142,124 new triples in addition to the 96,108 unique CORD-19 triples and the density of the KG increased 2.48 times from 0.0408 to 0.101. Therefore, the final KG includes information from recently published literature (CORD-19), as well as decades of accumulated biomedical knowledge on genes, proteins, diseases, and phenotypes. All these relationships are saved into a format that can be directly imported into Neo4j, so that we can visualize the relationships and perform more complex graph queries using the graph query engines.

The fifth and final stage involved formatting for the KG nodes and edges for import into the Neo4j graph database. All triples data, from CORD-19 and other databases, were formatted into a common data format. This required creating a file listing the nodes in the KG and a file listing the edges in the KG, where each edge represents a specific instance of a triple in CORD-19 or other data source, along with the ID of the supporting text if the triple came from CORD-19, in order to retrieve the supporting texts if a unique triple is queried (Fig. 2). In total, there are 588,531 nodes and 1,122,436 edges, including text and article nodes and edges, in the final KG to be imported into Neo4j.

### Demonstration of web application user interface and functionality

**Fig. 3:**
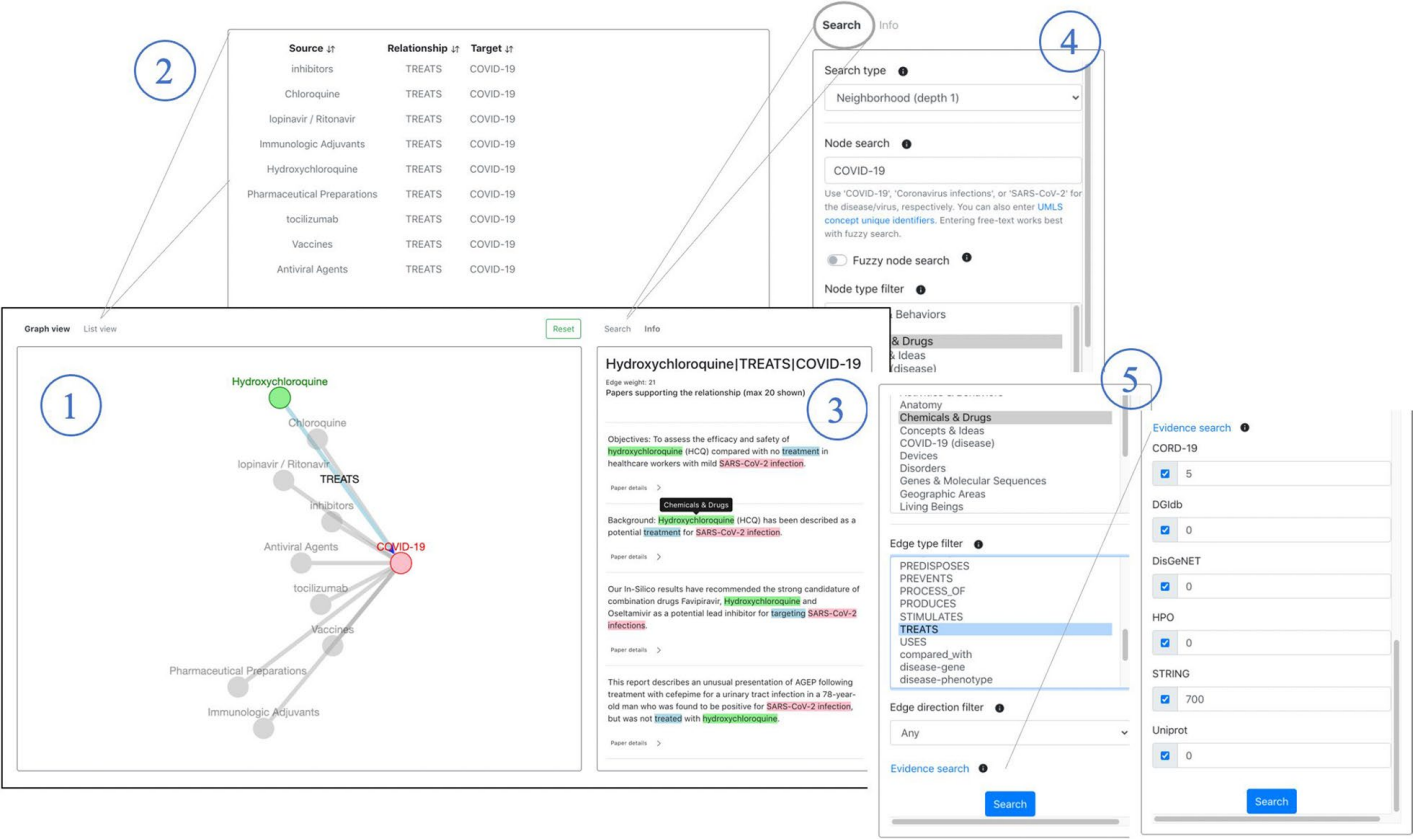
Web application user interface. (1) The left panel is a graph view that shows the nodes and edges returned from a search. Nodes and edges are labeled and color-coded for an effective user experience. A node can be clicked on to return more information on the entity, such as its UMLS Concept Unique Identifier (CUI), semantic group(s), and synonyms. Clicking an edge will bring up information on the triple, including the supporting texts, in the panel on the right, which the image demonstrates. (2) In addition to viewing the search results as a graph, one can use the list view to view the same nodes and edges in a table, which can be sorted. (3) The right panel will show information on the triple that was clicked on. The head, relationship, and tail of the triple are color-coded in the text for easy interpretation. Metadata information of the text can be seen by clicking “Paper details”. Hovering over highlighted text shows more information about the entity. (4) The right panel also has a tab to do the actual search. One can select the type of search, the node to search for, whether or not to use fuzzy search, what node filters to use, as well as (5) what edge filters to use, the edge direction, what data sources to use, and the confidence level (i.e. edge weight) required for each data source

A web application was designed to serve as the frontend for visualizing and querying the KG (Fig. 3), with features to make the user experience as fluid as possible. To make viewing information easier, both a graphical view displaying nodes and edges as well as a list view showing relationships were added. Additionally, interacting with edges and relationships allows the user to explore articles that support that relationship. To allow the user to filter the data, a search feature was implemented with filters based on node labels, edge labels, node types, evidence sources, and relationship confidence for each source (i.e. edge weight). These features allow the user to tune the KG based on the entities and relationships of interest, as well as desired data/evidence sources, while setting a threshold to their strength of evidence. While the KG web application can be used for a breadth of purposes, in this section we will highlight three different types of searches and their use cases below. These searches and their results can be replicated by following the tutorial section of the KGEV website (http://covid19nlp.wglab.org/tutorial).

**Fig. 4:**
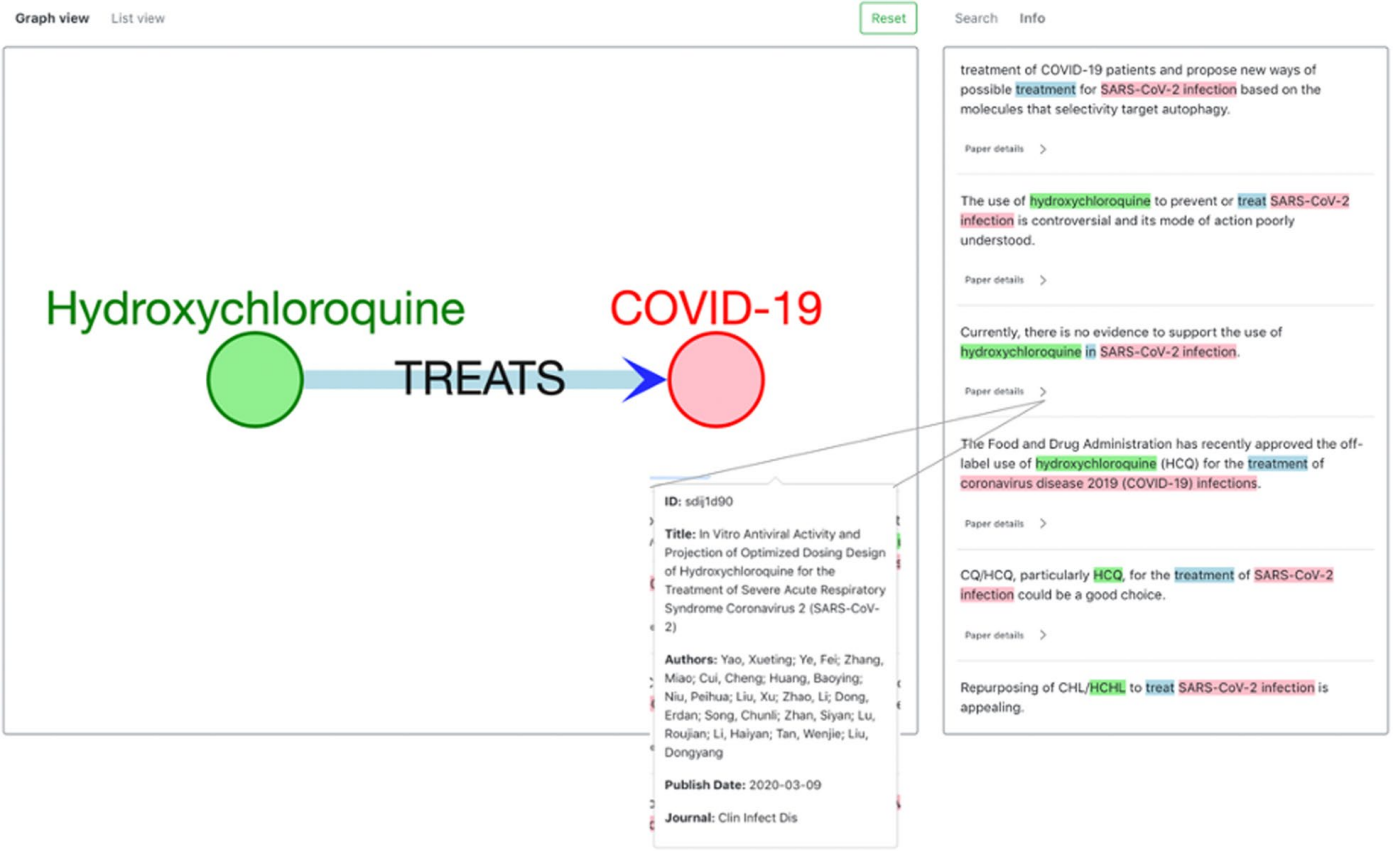
Direct search example. In this example, one may want to find articles supporting, disproving, or mentioning that hydroxychloroquine can be used to treat COVID-19. The panel on the right shows texts mentioning this relationship, and colors are used to make it obvious where the triple appears in the text, so it can be quickly validated by the user. By clicking on “Paper details” for any text, metadata information, including the article ID (CORD-19 ID), title, authors, publish date, and journal, will be shown as the figure demonstrates. The panel on the left shows the triple as a direct relationship between the two entities, and the head, relationship, and tail are all labeled

The first use case is a direct search, that is, finding evidence that directly mentions a relationship between two entities. In this case, the user wants to retrieve texts mentioning a relationship between two known entities. For example, a user may want to find literature on the use of hydroxychloroquine to treat COVID-19. They can use the web application user interface to query the triple hydroxychloroquine—> TREATS—> COVID-19 (Fig. 4). Upon clicking on the edge between the two nodes, the user interface shows texts where this relationship exists. The idea is that the supporting texts provide a brief summary of the relationship, and the user can be redirected to the full articles through the metadata pop-up. In this example, while some of the supporting texts say that repurposing hydroxychloroquine to treat COVID-19 is appealing, there are texts that say there is no evidence to support its use and that it is controversial and poorly understood. The texts serve only to summarize where the triple was mentioned and retrieve relevant articles but may not provide the complete picture; a user can click “Paper details” and be redirected to read the full article in detail.

**Fig. 5:**
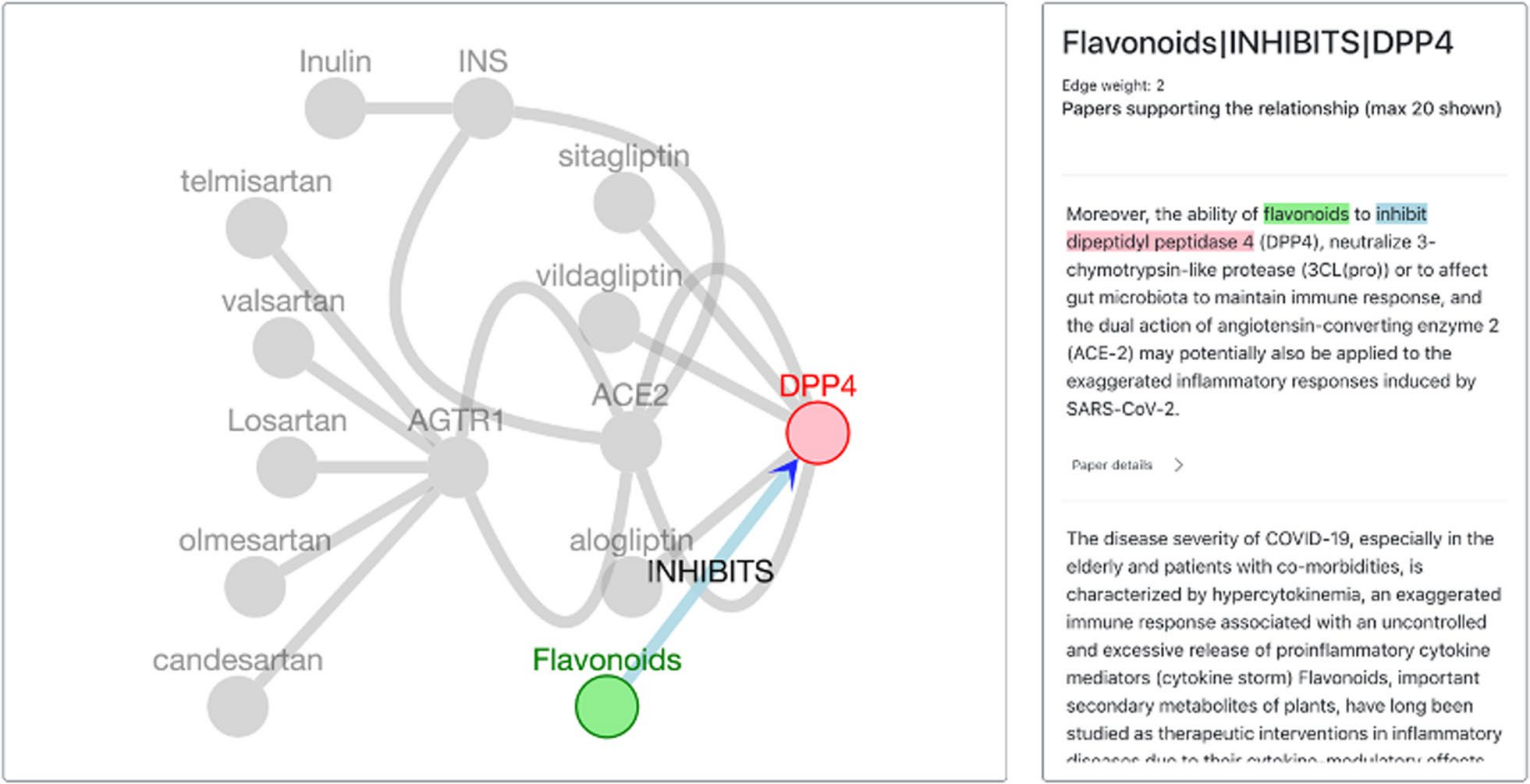
Neighborhood search example. A neighborhood search with a depth of two was performed using ACE2 as the start node. The neighbors of ACE2 were filtered so they only represent genes that have a gene–gene relationship in STRING with an interaction score >  = 700, representing high confidence interactions. The second layer of edges represent drug-gene interactions from DGIdb (interaction score >  = 2) and CORD-19 (at least two sources of evidence). The right panel shows support texts for flavonoids inhibiting DPP4, a gene that interacts with ACE2. The graph in the figure represents a subset of the results; The full results can be viewed by following the search settings in the tutorial section of the KGEV website (http://covid19nlp.wglab.org/tutorial)

While it is useful to obtain texts and articles mentioning relationships of interest, the user may only have an idea of what to search for but not the exact triple. The second use case is a neighborhood search, which would be helpful in this case. Using neighborhood search, a user can obtain the neighborhood of a node, that is, all the nodes connected to the search node by an edge. Additionally, the neighborhood search can be run at a depth of two to include neighbors of neighbors. An example use case is if a user wants to explore possible treatments for COVID-19 generally rather than a specific drug. Using neighborhood search, the user can search for drugs that interact with genes that interact with ACE2, since SARS-CoV-2 uses ACE2 for host cell entry [40]. Various filters in the user interface can be used to accomplish this search. This example search returns some drugs like sitagliptin, vildagliptin, and alogliptin that are DPP4 inhibitors (Fig. 5). While DPP4 inhibitors have been tested as a treatment for COVID-19, the results are still inconclusive [41]. From CORD-19 we also extracted the relationship that flavonoids inhibit DPP4 as well, and the supporting text seems to suggest that it may be used to reduce the SARS-CoV-2 inflammatory response. This example shows how KGEV, with its integration of gene–gene relationships, gene-drug relationships and relationships from literature, can be used for knowledge exploration and hypothesis generation.

**Fig. 6:**
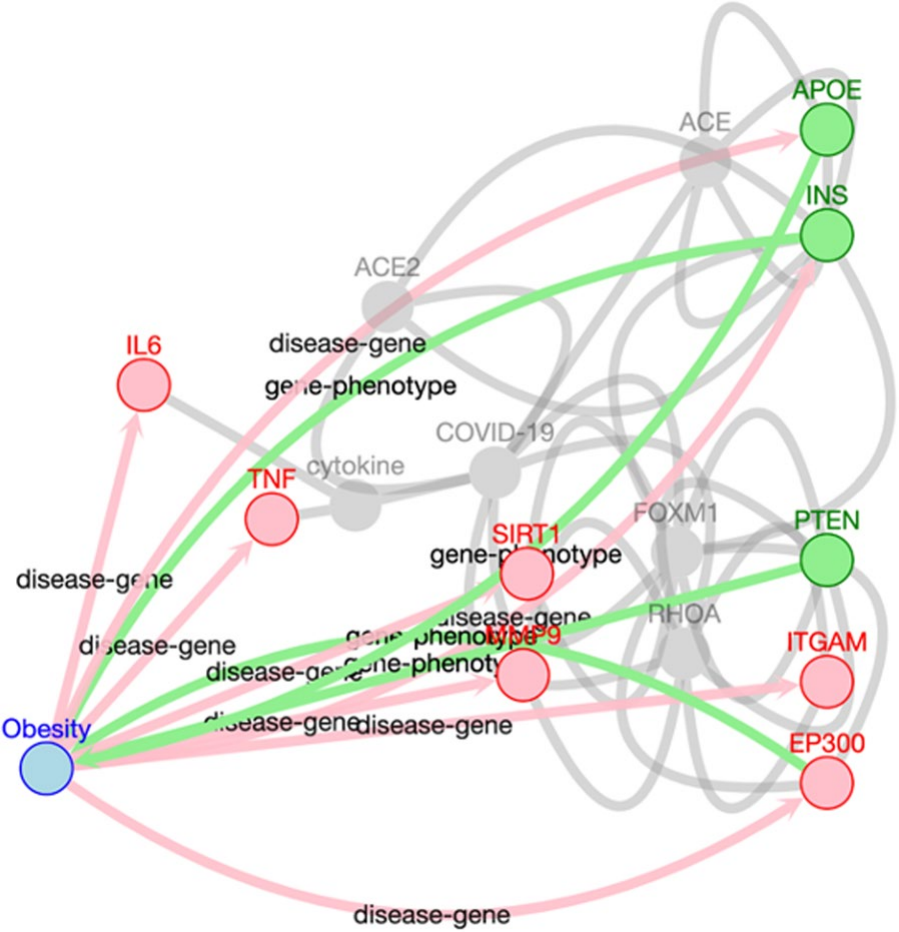
Shortest path search example. The search represents paths with a length of three edges between obesity and COVID-19 through nodes under the UMLS semantic group of “Genes & Molecular Sequences”. Direct paths are excluded. The edges represent disease-gene relationships from DisGeNET and gene-phenotype relationships from HPO to obtain obesity-associated genes, and gene–gene relationships from STRING (interaction score >  = 700, representing high confidence interactions) and CORD-19 (at least 7 supporting texts). The search results represent some of the pathways through with obesity may be associated with COVID-19 risk. The graph in the figure represents a subset of the results; The full results can be viewed by following the search settings in the tutorial section of the KGEV website (http://covid19nlp.wglab.org/tutorial)

In addition to direct paths and neighborhood search, a user may be interested in exploring the relationship between two entities in more detail. The third and last type of search, shortest paths search, would be useful in this situation. In this case, the user is interested in understanding what entities mediate the relationship between two entities of interest. An example use case is to use the shortest paths search through the web application user interface to find the pathways that are shared by obesity and COVID-19 (Fig. 6). The shortest path search function does not necessarily need to return exclusively the shortest path; it is also possible to return paths within a given length, for example, one to three edges long. An option for excluding direct paths is also available. Additionally, the nodes in the middle of the path can be filtered to look for certain types of mediators between the two entities of interest; in this example we only allow genes within the shortest path. The shortest paths result for this example shows that pathways such as immune response may mediate the relationships between obesity and COVID-19. One could hypothesize that obesity negatively affects the innate immune response, which predisposes COVID-19, and there is some research in this area [42]. Again, we obtain useful information and formulate new hypothesis through the integration of gene-phenotype, disease-gene, and gene–gene relationships from databases and literature.

### Turn-key adaption of the KGEV framework to study phenotypic presentations of human diseases

**Fig. 7:**
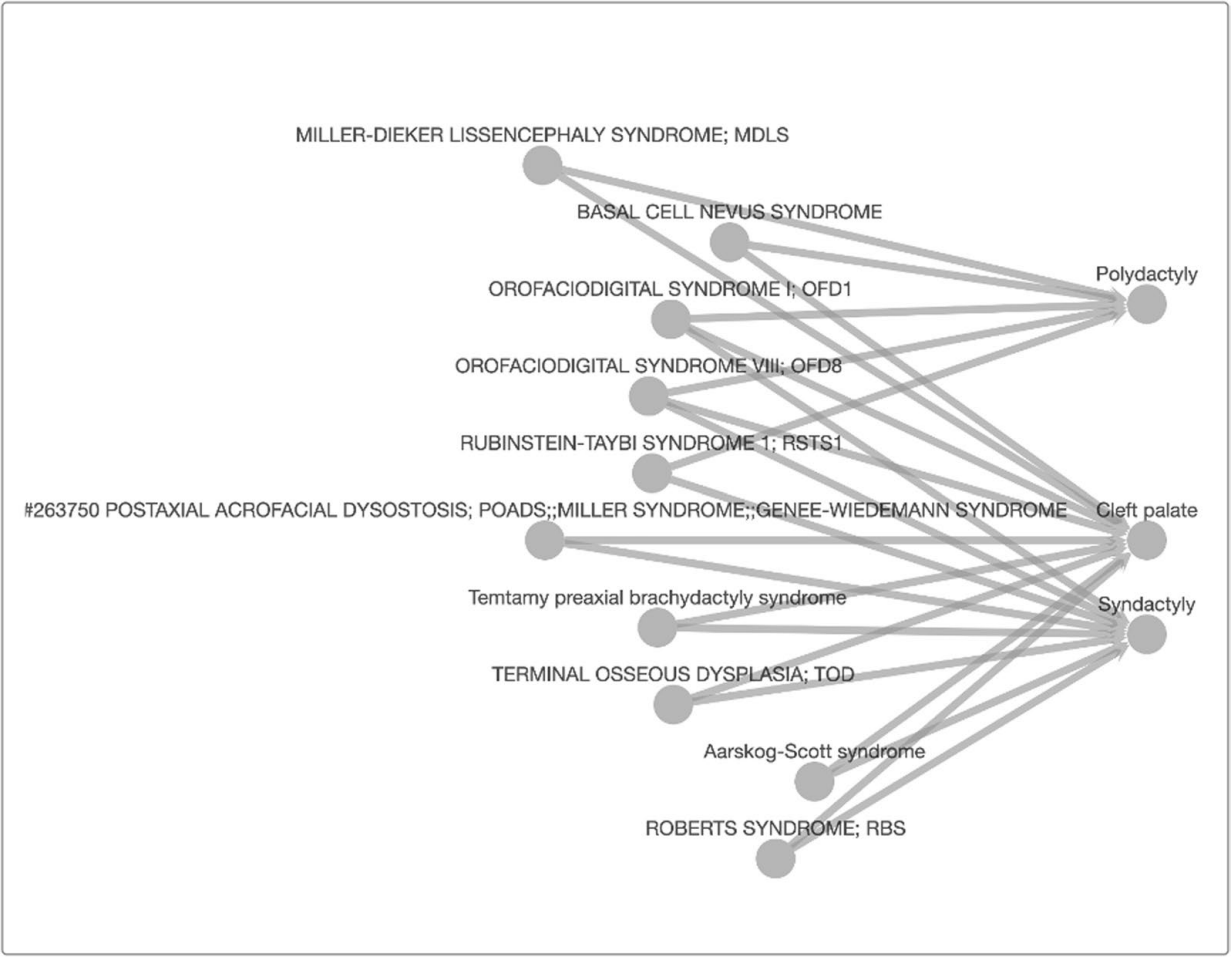
Turn-key adaptation of KGEV to study human disease phenotypes. The entire HPO database of disease-phenotype relationships was stored in a new Neo4j database, following the schema of the COVID-19 database (but without text information). Just by swapping the database, one can use the same user interface to query the KG, but on a different set of data. The figure shows an example of finding the shortest path between polydactyly, syndactyl, and cleft palate phenotypes to determine diseases that share at least two of the three phenotypes

Although we used COVID-19 as a use case above, the design of the KGEV web framework is flexible, so that it can be immediately repurposed to study other biomedical domains. To illustrate this, we took the annotation database from the Human Phenotype Ontology (HPO), which links genes to clinical phenotypes and disease names. We then created a new graph database in Neo4j and re-connected the web application to this new graph database via changing a simple parameter that indicates the database URL and port. As we showed in Fig. 7, the web application can immediately display entity relationships in HPO, and users can immediately start searching biomedical questions such as “what are the diseases that have at least two of the three phenotypes: polydactyly, syndactyl, and cleft palate”. The query returns a list of clinical diseases (based on HPO) and displays them in a graphical format. While we recognize that the HPO website (https://hpo.jax.org/app/) already includes the same functionality to search phenotypes for a given human disease, the search functionality in KGEV also allows users to easily find diseases sharing two or more phenotypes, or phenotypes shared by two or more diseases. This example demonstrates the versatility of the KGEV framework that can be easily adapted to many other general purposes.

## Discussion

Knowledges graphs (KGs) serve as a convenient framework for structuring knowledge by reducing information into a set of entities and their relationships. A number of computational methods have been developed to generate KGs from biomedical literature (for example, through natural language processing), and some researchers have leveraged the generated KGs for downstream tasks such as link prediction, neighborhood search, and question answering. However, there is a general lack of computational tools or web frameworks to support the exploration and visualization of the KG themselves, which would facilitate interactive knowledge discovery and formulation of novel biological hypothesis. In this study, to address this issue, we created a framework for Knowledge Graph Exploration and Visualization (KGEV), that allows researchers to explore a KG interactively, search for specific entities and relationships from multiple data sources, and quickly access information along with supporting papers. Unlike other KGs, our framework allows fast retrieval of relevant texts supporting the relationships in the KG, thus allowing human reviewers to judge the reliability of knowledge extracted. Beyond the utility of the visualization framework, the interactive web application will aid researchers in understanding the work that has been done thus far while helping guide questions for future research projects. Our KG can prioritize: (*1*) primary relationships between biomedical entities, which are directly extracted from free unstructured text by the NLP approach, and (*2*) secondary relationships from existing knowledgebases, inferred from a variety of biomedical databases on genes, proteins, phenotypes, drugs, and diseases. Our incorporation of greatly expanded inputs, as compared with other studies, further allows for the comprehensive identification of known and hidden knowledge for use in subsequent steps.

Although we used COVID-19 as a case study here, the computational tools developed as part of the protocol can be applied to a broad range of emerging diseases in the future, to quickly gather scientific evidence from a sudden surge of literature information. We recognize that many COVID-19 literature mining web servers, such as the NIH’s LitCOVID [43] and Google’s COVID-19 Research Explorer (https://covid19-research-explorer.appspot.com/), were developed to provide literature searching system and there exist COVID-19 KG web servers [20, 25, 26]. However, these tools are of limited use for researchers who need the flexibility to both explore the nodes and edges of the KG directly and the ability to quickly retrieve relevant biomedical texts, as well as open-source pipelines and resources to customize the knowledge graph and web application for their own needs.

While the KG visualization framework facilitates data exploration and the retrieval of relevant biomedical literature, there are some limitations to this framework. First, the quality of the KG exploration and visualization depends on the quality of the data. The KG generation was relatively simple in our case study. While SemRep is a publicly accessible tool that is commonly used for relationship extraction, the relationships extracted by SemRep might not be optimized for a specific domain, such as COVID-19 [29, 44]. However, our framework is flexible enough that users can use other NLP tools to perform the relationship extraction from biomedical literature. Additionally, KG validation and refinement remain a challenge [45]. While the focus of KGEV is KG exploration and visualization rather than construction, the integration of data from alternative sources may lead to different relationships in the KG. For example, 13,962 gene-disease relationships from DisGeNET (curated gene-disease associations) were integrated into the COVID-19 KG while 709,156 gene-disease relationships were integrated when using all gene-disease associations in the Comparative Toxigenomics Database (CTD) [46], including 12,325 relationships overlapping with DisGeNET (Additional file 1: Table S2). KGEV does offer some tools for KG validation. With the ability to filter by confidence level per data source, the confidence level being provided by the data source or equal to the number of supporting texts from literature, one can adjust the rate of false positive edges in the KG. Additionally, one can use the KGEV pipeline to quickly and mechanistically construct and test KGs using alternative data sources.

Another limitation of the framework is that it requires users to have some a priori hypothesis or question in order to know what to search for in the KG. However, neighborhood and shortest path search can identify new nodes and links of interest for further exploration. Finally, while the web application can help answer simple questions through triple retrieval, such as “what treats COVID-19”, it may be more difficult to answer questions where the answer involves multiple entities and relationships that are complex and dependent on each other. This issue can be addressed by improved approaches to translate user questions in the web interface into appropriate graph database queries for the Neo4j backend. Going forward, it may also be useful to visualize algorithms and their output that are run on top of the KG, like node clustering, link prediction, question answering, and allow for custom KG queries.

Despite the limitations discussed above, our KGEV framework can adequately describe a range of important biomedical entities associated with diseases, relate phenotypes to genes to drugs using contextual relationships, and thereby inform hypothesis generation and, ultimately, personalized treatment approaches for other emerging disease.

## Conclusions

In an era of literature explosion, the KGEV framework can be applied to many emerging diseases to support structured navigation of the vast amount of newly published biomedical literature and other existing biological knowledge in various databases. It can be also used as a general-purpose tool to explore and query gene-phenotype-disease-drug relationships interactively.

## Supplementary Information

**Additional file 1: Table S1.** A list of normalized COVID-19/SARS-CoV-2-related subjects. **Table S2. **COVID-19 KG data source comparison.

**Additional file 2: Fig. S1. **COVID-19 KG schema. The five nodes represent the five major entity groups in the COVID-19 KG (PHENO=PHENOTYPE, GO=GENE ONTOLOGY) and the edges are color-coded based on the data sources supporting relationships between the two connected nodes. Each node in the KG is uniquely identified by its node label, which is based on the SemRep outputted “preferred name”, gene symbol for genes, and dictionary-based entity standardization for COVID-19-/SARS-CoV-2-specific terminology (Additional file 1: Table S1). A node can have multiple related identifiers (i.e. Concept Unique Identifiers or CUIs) and node types (i.e. UMLS semantic group) depending on context. Example KG nodes are shown in the table in the figure.

## Data Availability

A demo web server on COVID-19 can be accessed at http://covid19nlp.wglab.org. The web application can be deployed as two Docker containers, one for the backend application and one for the frontend application, which are available, respectively, at https://hub.docker.com/u/genomicslab/kgev-backend and https://hub.docker.com/u/genomicslab/kgev-frontend. Code used to integrate relationships from SemRep and other data sources described in Methods, links to these data sources, and code to format the data for Neo4j import can be found at https://github.com/WGLab/kgev-neo4j. The COVID-19 Open Research Dataset (CORD-19) is available publicly at https://www.kaggle.com/allen-institute-for-ai/CORD-19-research-challenge.

## Abbreviations

AI: Artificial intelligence
CORD-19: COVID-19 open research dataset
CUI: Concept unique identifier
GO: Gene ontology
HPO: Human phenotype ontology
KG: Knowledge graph
KGEV: Knowledge Graph Exploration and Visualization
NLP: Natural language processing
UMLS: Unified Medical Language System
