# Supplementary figures and images for: Expediting knowledge acquisition by a web framework for Knowledge Graph Exploration and Visualization (KGEV): case studies on COVID-19 and Human Phenotype Ontology
Source: BMC Med Inform Decis Mak. 2022 Jun 2;22(Suppl 2):147. doi: 10.1186/s12911-022-01848-z (PMC9161770; doi:10.1186/s12911-022-01848-z)

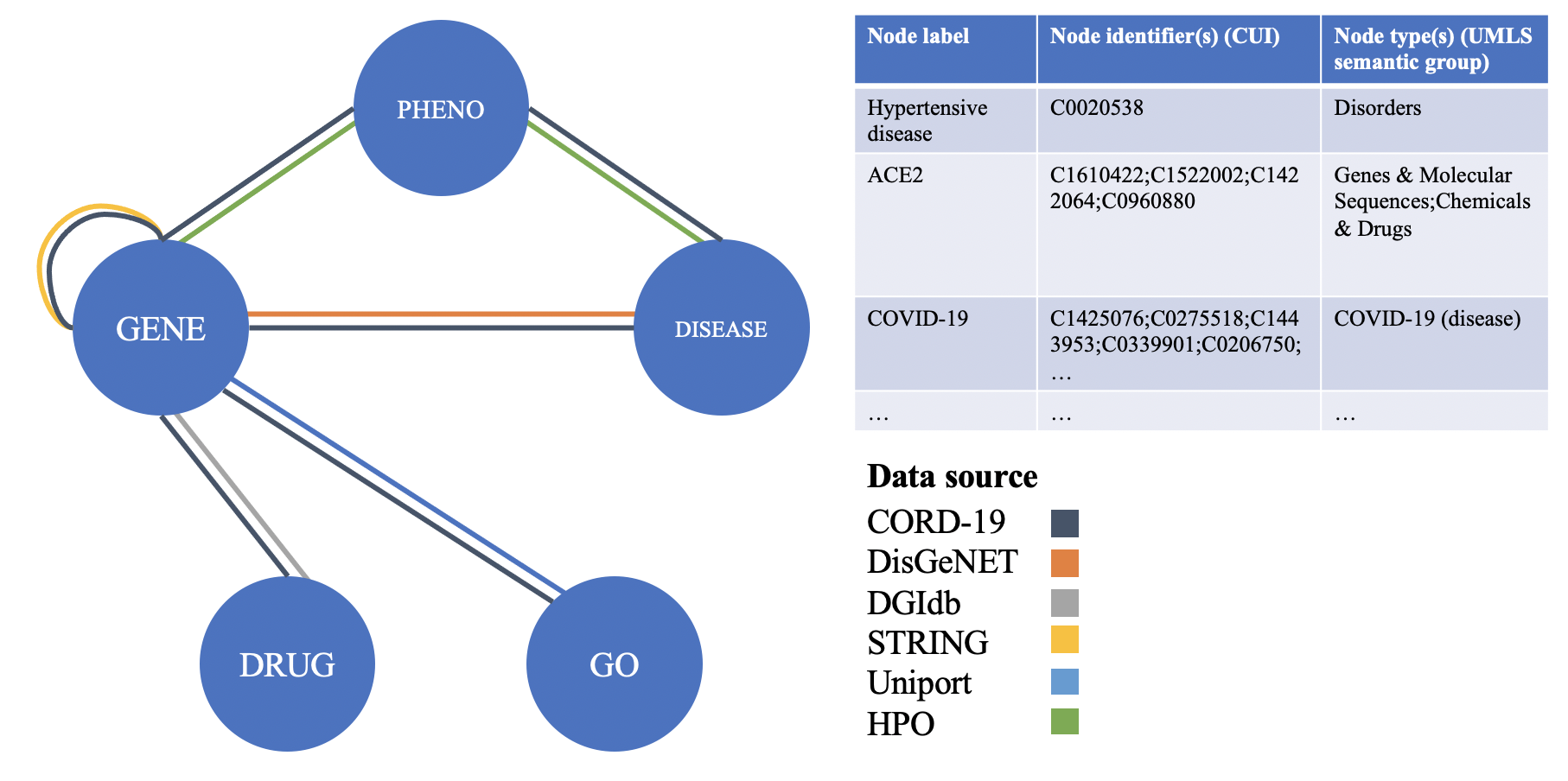

Supplement: Supplementary file 2 — Additional file 2: Fig. S1. COVID-19 KG schema. The five nodes represent the five major entity groups in the COVID-19 KG (PHENO=PHENOTYPE, GO=GENE ONTOLOGY) and the edges are color-coded based on the data sources supporting relationships between the two connected nodes. Each node in the KG is uniquely identified by its node label, which is based on the SemRep outputted “preferred name”, gene symbol for genes, and dictionary-based entity standardization for COVID-19-/SARS-CoV-2-specific terminology (Additional file 1: Table S1). A node can have multiple related identifiers (i.e. Concept Unique Identifiers or CUIs) and node types (i.e. UMLS semantic group) depending on context. Example KG nodes are shown in the table in the figure. [file 12911_2022_1848_MOESM2_ESM.png]
